# Association of the triglyceride glucose index with all-cause and cardiovascular mortality in a general population of Iranian adults

**DOI:** 10.1186/s12933-024-02148-8

**Published:** 2024-02-12

**Authors:** Ghazaal Alavi Tabatabaei, Noushin Mohammadifard, Hamed Rafiee, Fatemeh Nouri, Asieh Maghami mehr, Jamshid Najafian, Masoumeh Sadeghi, Maryam Boshtam, Hamidreza Roohafza, Fahimeh Haghighatdoost, Marzieh Taheri, Nizal Sarrafzadegan

**Affiliations:** 1 Isfahan Cardiovascular Research Center, Cardiovascular Research Institute, Isfahan University of Medical Sciences, P. O. Box: 81745-15, Isfahan, Iran; 2 Interventional Cardiology Research Center, Cardiovascular Research Institute, Isfahan University of Medical Sciences, Isfahan, Iran; 3 Pediatric Cardiovascular Research Center, Cardiovascular Research Institute, Isfahan University of Medical Sciences, Isfahan, Iran; 4 Department of Statistics, Yazd University, Yazd, Iran; 5 Hypertension Research Center, Cardiovascular Research Institute, Isfahan University of Medical Sciences, Isfahan, Iran; 6 Cardiac Rehabilitation Research Center, Cardiovascular Research Institute, Isfahan University of Medical Sciences, Isfahan, Iran; 7 Heart Failure Research Center, Cardiovascular Research Institute, Isfahan University of Medical Sciences, Isfahan, Iran; 8 Faculty of Medicine, School of Population and Public Health, University of British Columbia, Vancouver, Canada

**Keywords:** Insulin resistance, Triglyceride-glucose index, All-cause mortality, Cardiovascular mortality

## Abstract

**Background:**

The triglyceride glucose (TyG) index is a new and low-cost marker to determine insulin resistant which may be a predictor of cardiovascular disease (CVD). Although available evidence showed that its association with CVD mortality (CVM) and all-cause mortality (ACM) may differ in different populations, scarce data are available in this regard specially in low and middle-income countries.

**Purpose:**

To examine the association between TyG index and risk of CVM and ACM in Iranians.

**Methods:**

This prospective cohort study included 5432 adults (age ≥ 35 years) with no history of CVD events. Fasting glucose and triglyceride were measured at baseline in all participants and TyG index was calculated. Cox frailty model was used to calculate hazard ratios (HRs) for CVM and ACM across the tertiles of TyG index.

**Results:**

After a median follow-up of 11.25 years, a total number of 191 cardiovascular deaths, and 487 all-cause mortality was recorded. The risk of both CVM and ACM increased across the tertiles of TyG index. In the adjusted model for lifestyle and metabolic variables, the risks of ACM and CVM increased by 41% (95% CI 1.11, 1.81; P for trend = 0.005) and 64% (95% CI 1.07, 2.50; P for trend = 0.024), respectively. However, adjustment for diabetes mellitus disappeared the significance for both ACM and CVM. These associations may vary by sex. TyG was not related to the risk of non-CVD mortality.

**Conclusion:**

The predicting value of TyG index for ACM and CVM might be mediated by diabetes status. Further studies are required to confirm these findings.

**Supplementary Information:**

The online version contains supplementary material available at 10.1186/s12933-024-02148-8.

## Introduction

Coronary artery disease (CAD), peripheral artery disease (PAD), and stroke are all regarded to be forms of cardiovascular disease (CVD). CVD and its ensuing complications make up for a significant economic burden and the leading cause of mortality in developed countries and increasingly so in low and middle-income countries [1]. In the following decades, cardiovascular mortality is expected to escalate by 137% and 120% in males and females, respectively [2]. Identifying those who are more susceptible to CVD in its early stages and improving their lifestyles has been proposed to surmount these problems.

Insulin resistance (IR) is identified as the leading culprit of metabolic abnormalities connected to CVD [3]. IR is characterized by impaired responsiveness of peripheral tissue to insulin and is linked to increased mortality and morbidity [3, 4]. A previous meta-analysis reported that IR is independently associated with a higher risk of CVD and all-cause mortality in the non-diabetic individuals [5]. Traditionally, hyper insulinemic-euglycemic (HIEC) clamp test and the homoeostasis model assessment of insulin resistance (HOMA-IR) have been used to measure IR. However, in recent years, triglyceride glucose index (TyG) has been presented as a more convenient and reliable IR surrogate than HIEC and HOMA-IR [6].

A series of recent studies have indicated that elevated TyG index is linked to a higher risk of CVD [7], CAD [8], PAD [9], and stroke [10]. Nevertheless, the relationship between TyG index and CVD mortality (CVM) and all-cause mortality (ACM) is still obscure. In old hypertensive individuals (> 60 y), a nine-linear association was observed between TyG and ACM while the lowest risk was found at the level of 9.45 and values lower and higher than 9.45 were associated with increased incidence risk of ACM [11]. This study failed to find any significant association between TyG and CVM. A recent systematic review and meta-analysis of twelve cohort studies demonstrated an association between TyG index and CAD, CVD, and myocardial infarction (MI); but no statistically significant relationship was found between TyG index and CVM and ACM [6].

Low- and middle- income countries (LMIC) has experienced a remarkable rise in the frequency of CVD and CVM in recent decades [12]. Eighty percent of CVM cases in recent years have been reported in LMIC, particularly in Middle Eastern countries [7, 8]. Furthermore, studies have demonstrated that Iranians exhibit a greater prevalence of risk factors for CVD, central obesity [13], metabolic syndrome [14], and diabetes mellitus (DM) [15] when compared to the general population of other countries[16, 17] that indicate impaired metabolism and hyperinsulinemia [18, 19].

Despite the importance of identifying individuals from the general population with increased risk of CVD incidence and accompanying mortality, the literature on predictive value of the TyG index regarding CVM and CAM is inconsistent. Therefore, we aimed to investigate whether a higher TyG index in the general Iranian population can predict the risk of cardiovascular and all-cause death.

## Methods

### Study population

The ICS is a population-based study that included 6504 people with ages equal to or greater than 35 years old (3168 men and 3336 women). Participants had no prior CVD at baseline and were chosen from 3 cities in Iran (Isfahan, Arak, Najaf-Abad).

**Fig. 1:**
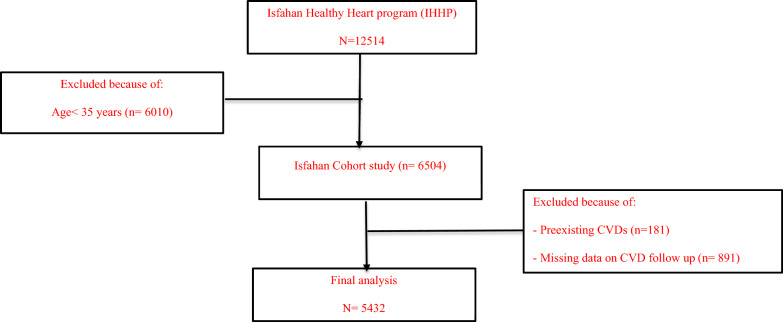
Folow of participants through the study

The study design has already been described in further details [20]. Initially, the researchers collected information on lifestyle factors, such as diet, through in-person interviews and followed up with participants every two years to monitor specific outcomes. If no cardiovascular events occurred during these follow-ups, all variables, including lifestyle factors, were reassessed in a subsequent survey after six years (in 2007 and 2013). In the present analysis, we enrolled participants who had complete information on their medical status, triglyceride, glucose and relevant confounding factors. Each individual involved in the study gave their written consent after being informed about the details of the study. The research adhered to the guidelines laid out in the Declaration of Helsinki. The Ethic Committee of the Research Council of the Isfahan Cardiovascular Research Center, a World Health Organization partner facility in Isfahan, Iran (#1402114), gave its approval to this study. Figure 1 illustrates the flowchart of our study selection process, outlining the inclusion of patients and providing reasons for the exclusion of other participants.

### Data collection

A 30-min interview at baseline was performed to collect data on demographic and socioeconomic factors. Patients were also asked about their medical (including dyslipidemia, DM, HTN, and medications) and lifestyle factors.

Height, weight, systolic (SBP) and diastolic blood pressure (DBP) were measured during physical examination. A12 h fasting blood sample was gathered from all participants. The samples were analyzed at the central laboratory of the Isfahan Cardiovascular Research Center, following strict quality control measures set by national and international standards. An autoanalyzer made by Eppendorf in Hamburg, Germany was used to measure levels of FPG, TG, and total cholesterol through enzymatic methods. To measure HDL-C, low-density and very low-density lipoproteins were removed from the blood using dextran sulfate magnesium before analysis [21]. Diabetes was characterized by a fasting plasma glucose (FPG) equal to or exceeding 126 mg/dL, a 2-h postprandial glucose (2hpp) equal to or exceeding 200 mg/dL, or the use of anti-diabetic medications [22]. The global dietary index (GDI) was determined by assessing food quality. 29 food items were grouped into seven categories and given scores of 0, 1, or 2 based on how often they were eaten. Healthier food groups received higher scores while unhealthy ones received lower scores. The scores for all seven categories were added to calculate the GDI, with lower values indicating a healthier diet. Weight (Kg) divided by square of height (m2) was used to compute the body mass index (BMI). The TyG index was calculated as Ln (fasting TG (mg/dl) × FPG (mg/dl)/2) [23]. Individuals with SBP ≥ 140 mmHg and\or DBP ≥ 90 or those who were taking antihypertensive drug were considered HTN. Dyslipidemia was described as if LDL-C ≥ 130 mg/dL, TC ≥ 200 mg/dL, TG ≥ 150 mg/dL or HDL-C < 40 mg/dL in men or < 50 mg/dL in women.

### Follow-up

Participants were interviewed biannually over the phone. Data regarding mortality was collected by conducting verbal autopsies with family members who survived the deceased individual. The approach involved a structured primary interview wherein the initial inquiry was whether the person in question was still alive. The study under consideration defined cardiovascular mortality as the occurrence of fatal myocardial infarction, fatal strokes (i.e., death resulting from cerebrovascular disease), and sudden cardiac death. For the purposes of the study, sudden cardiac death was characterized as death occurring within one hour after onset, witnessed cardiac arrest, or abrupt collapse without preceding symptoms lasting longer than 41 h.

### Statistical analysis

Participants were categorized into three groups based on the tertile of TyG index. We compared the baseline characteristics of male and female participants using independent sample t-tests (or Mann–Whitney tests if necessary) for continuous variables and chi-square tests for categorical variables. We also compared the baseline characteristics of participants across the tertiles of the TyG index using analysis of variance (ANOVA) (or Kruskal–Wallis tests if assumptions were not met) and chi-square tests, for continuous and categorical variables, respectively. We presented continuous variables as mean ± SD and categorical variables as number (percent).

We calculated person-years of follow-up from recruitment until the occurrence of death. We calculated crude and multiple-adjusted hazard ratios (HRs) and 95% confidence intervals (CIs) to determine the association between the TyG index and mortality using Cox proportional hazards regression. Model 1 was unadjusted, and model 2 was adjusted for age at baseline and sex. Model 3 included additional adjustments for education, marital status, and residency area (rural vs. urban). Model 4 included adjustments for GDI, smoking status, and total daily physical activity. The final model further adjusted for BMI, hypertension, and elevated total cholesterol. We additionally controlled the mediating effect of diabetes in model 6. All statistical analyses were performed using SPSS (v. 26) and STATA (v 14.0), considering a p ≤ 0.05 to be statistically significant.

## Results

During a median follow-up of 11.25 years, 487 cases of ACM (overall incidence of 7.76 per 1000 person-years), 181 cases of CVM (overall incidence of 2.88 per 1000 person-years) and 306 cases of non-cardiovascular mortality (overall incidence of 4.88 per 1000 person-years) were recorded. There was no evidence for interaction between diabetes and TyG either in the whole population or in men and women separately.

Baseline characteristics of participants according to the tertiles of TyG index is illustrated in Table 1. Participants in the higher TyG index group were older than the first group (51.75 ± 11.08 vs. 49.38 ± 11.88; p < 0.001) and were more likely to be physically inactive, live in rural areas, and have lower diet quality (GD). Moreover, HTN, DM, dyslipidemia, and overweight (BMI ≥ 25) were more frequent in individuals in the third tertile of the TyG index compared with the first one (p < 0.001). SBP (126.59 ± 21.61 vs 117.04 ± 19.64; p < 0.001) and DBP (80.72 ± 11.74 vs 75.86 ± 10.70; p < 0.001) were higher in participants in the third tertile of the TyG index in comparison with the first one. Similar trends were observed for FPS, TC, HDL-c, LDL-c, TG and TG:HDL (p < 0.001).

**Table 1:** Baseline characteristics of the participants by tertiles of triglyceride glucose index

	Overall N = 4973	Triglyceride glucose index			P value^†^
		Tertile 1 (TyG < 8.58) N = 1810	Tertile 2 (8.585 ≤ TyG < 9.09) N = 1811	Tertile 3 (9.09 ≤ TyG) N = 1811	
Males, n (%)	2648 (48.7)	864 (47.7)	885 (48.9)	899 (49.6)	0.514
Age, years (Mean ± SD)	50.69 (11.62)	49.38 (11.88)	50.96 (11.77)	51.75 (11.08)	< 0.001
Education					0.923
≤ 5 years	3861 (71.2)	1290 (71.4)	1277 (70.6)	1294 (71.6)	
6–12 years	1230 (22.7)	411 (22.7)	419 (23.2)	400 (22.1)	
≥ 13 years	333 (6.1)	106 (5.9)	113 (6.2)	114 (6.3)	
Married, n (%)	4951 (91.1)	1665 (92.0)	1659 (91.6)	1627 (89.8)	0.052
Urban, n (%)	1501 (27.6)	565 (31.2)	503 (27.8)	433 (23.9)	< 0.001
Physical activity (MET.min/wk) (Mean ± SD)	872.38 (548.62)	898.53 (548.11)	883.33 (557.70)	835.28 (538.19)	0.001
Global dietary index	1.02 (0.27)	1.06 (0.24)	1.02 (0.27)	0.98 (0.28)	< 0.001
Smoking status, n (%)					0.628
Current	292 (16.1)	267 (14.7)	287 (15.8)	846 (15.6)	
Former	353 (6.5)	120 (6.6)	110 (6.1)	123 (6.8)	
Never	4233 (77.9)	1398 (77.2)	1434 (79.2)	1401 (77.4)	
Hypertension, n (%)	1510 (27.8)	344 (19.0)	514 (28.4)	652 (36.0)	< 0.001
Diabetes, n (%)	459 (8.4)	16 (0.9)	46 (2.5)	397 (21.9)	< 0.001
Dyslipidemia, n (%)	4735 (87.2)	1202 (66.4)	1729 (95.5)	1804 (99.6)	< 0.001
BMI (kg/m^2^) (Mean ± SD)	26.77 (4.68)	25.07 (4.46)	26.90 (4.56)	28.35 (4.43)	< 0.001
Waist to hip ratio	0.93 (0.08)	0.91 (0.09)	0.93 (0.08)	0.95 (0.08)	< 0.001
Systolic blood pressure, mm Hg	121.64 (20.95)	117.04 (19.64)	121.28 (20.44)	126.59 (21.61)	< 0.001
Diastolic blood pressure, mm Hg	78.40 (11.52)	75.86 (10.70)	78.61 (11.57)	80.72 (11.74)	< 0.001
Fasting glucose, mg/dL	88.67 (32.84)	76.83 (10.77)	81.79 (14.08)	107.38 (32.84)	< 0.001
Total cholesterol, mg/dL	214.12 (52.25)	187.86 (40.98)	212.57 (44.88)	241.91 (55.08)	< 0.001
LDL cholesterol, mg/dL	128.96 (43.42)	119.05 (38.31)	131.41 (42.22)	136.42 (47.39)	< 0.001
HDL cholesterol, mg/dL	46.91 (10.36)	47.99 (10.17)	46.59 (10.04)	46.16 (10.77)	< 0.001
TG, mg/dL	191.28 (103.26)	104.37 (24.90)	172.80 (33.00)	296.62 (106.24)	< 0.001
TG:HDL	4.31 (2.63)	2.29 (0.78)	3.88 (1.15)	6.77 (2.91)	< 0.001

Values are n (%) for categorical variables and mean (SD) for continuous variables

*BMI* body mass index, *LDL* low-density lipoprotein, *HDL* high-density lipoprotein, *TG* Triglycerides

^†^Derived from either an analysis of variance (ANOVA) or a Kruskal–Wallis test for continuous variables, and a chi-squared test for categorical variables

Table 2 highlights multivariate-adjusted HRs (and 95% CIs) for CVM, non-cardiovascular mortality and ACM across the tertiles of TyG index in the whole population. In the crude model, risk of ACM was higher in the highest tertile of the TyG index in the crude model (HR = 1.36, 95% CI 1.10, 1.69; P for trend = 0.005) and after adjustment for potential confounders in model 5 (HR = 1.41, 95% CI 1.11, 1.81; P for trend = 0.005). Similarly, individuals in the top tertile of TyG compared with those in the bottom tertile had 91% increased risk of CVM in the crude model (HR = 1.91, 95% CI 1.31, 2.78; P for trend = 0.001). Further adjustment for potential cofounding variables slightly weakened the association but did not affect its significance in model 5 (HR = 1.64, 95% CI 1.07, 2.50; P for trend = 0.024). However, there was no increased risk in non-cardiovascular mortality following the rise in the TyG index in crude model (HR = 1.13, 95% CI 0.86, 1.48; P for trend = 0.359). After full adjustment for potential confounding variables in model 5, there was a marginally significant increase in non-cardiovascular mortality in participants with the highest TyG index compared to those with those in the lowest tertile (HR = 1.32, 95% CI 0.97, 1.79; P for trend = 0.072). However, adjustment for diabetes as a mediator disappeared significance for both ACM (HR = 1.14, 95% CI 0.88, 1.49; P for trend = 0.329) and CVM (HR = 1.11, 95% CI 0.70, 1.75; P for trend = 0.732), and weakened the association for non-cardiovascular mortality (HR = 1.18, 95% CI 0.85, 1.64; P for trend = 0.318).

**Table 2:** Multiple-adjusted HRs (and 95% CIs) for all-cause, CVD and non-CVD mortality across tertiles of triglyceride glucose index

	Case/person year	Model 1	Model 2	Model 3	Model 4	Model 5	Model 6
All-cause mortality							
Tertile 1	140	1 (Ref)	1 (Ref)	1 (Ref)	1 (Ref)	1 (Ref)	1 (Ref)
Tertile 2	155	1.14 (0.90, 1.43)	1.06 (0.84, 1.33)	1.06 (0.84, 1.34)	1.09 (0.87, 1.38)	1.12 (0.88, 1.42)	1.09 (0.86, 1.40)
Tertile 3	192	1.36 (1.10, 1.69)	1.27 (1.02, 1.58)	1.28 (1.03, 1.60)	1.31 (1.05,1.64)	1.41 (1.11, 1.81)	1.14 (0.88, 1.49)
P for trend		0.005	0.029	0.026	0.015	0.005	0.329
Cardiovascular mortality							
Tertile 1	41	1 (Ref)	1 (Ref)	1 (Ref)	1 (Ref)	1 (Ref)	1 (Ref)
Tertile 2	61	1.53 (1.03, 2.27)	1.46 (0.98, 2.17)	1.47 (0.99, 2.18)	1.53 (1.03, 2.28)	1.38 (0.91, 2.10)	1.31 (0.86, 2.00)
Tertile 3	79	1.91 (1.31, 2.78)	1.82 (1.25, 2.66)	1.78 (1.21, 2.61)	1.85 (1.26, 2.71)	1.64 (1.07, 2.50)	1.11 (0.70, 1.75)
P for trend		0.001	0.002	0.003	0.002	0.024	0.732
Non-cardiovascular mortality							
Tertile 1	99	1 (Ref)	1 (Ref)	1 (Ref)	1 (Ref)	1 (Ref)	1 (Ref)
Tertile 2	94	0.97 (0.73, 1.29)	0.90 (0.68, 1.19)	0.90 (0.67, 1.19)	0.92 (0.69, 1.22)	1.00 (0.74, 1.36)	1.00 (0.74, 1.35)
Tertile 3	113	1.13 (0.86, 1.48)	1.04 (0.80, 1.37)	1.07 (0.81, 1.41)	1.10 (0.83, 1.44)	1.32 (0.97, 1.79)	1.18 (0.85, 1.64)
P for trend		0.359	0.732	0.603	0.511	0.072	0.318

Model 1: crude

Model 2: Adjusted for age (years) and sex (man/woman)

Model 3: Additionally adjusted for education level (0–5 years/6–12 years/ > 12 years), marital status (married/not married), residency location (urban/rural)

Model 4: Additionally adjusted for global dietary index (GDI), smoking status (never have smoked/have ever smoked), and total daily physical activity (METs-min/day)

Model 5: Additionally adjusted for BMI (kg/m^2^), hypertension (yes/no), and high total cholesterol (yes/no)

Model 6: Additionally adjusted for diabetes mellitus (DM) (yes/no)

Table 3 highlights the association between TyG index tertiles and mortality stratified by sex. In females, despite a significant positive association between the TyG index and ACM in the crude model (HR = 1.74, CI 1.23, 2.46; P for trend = 0.002), no statistically significant association was observed in any of the adjusted model. In males, higher TyG index was not associated with higher risk of ACM in the crude model (HR = 1.13, 95% CI 0.85, 1.50; P for trend = 0.378) but tended to be higher in model 5 after adjustment for various potential confounders (HR = 1.38, 95% CI 0.99, 1.92; P for trend = 0.056). however, adjustment for diabetes mellitus led to a null association (HR = 1.06, 95% CI 0.74, 1.51; P for trend = 0.760). Similarly, CVM was significantly higher in females in the top tertile of the TyG index in comparison with those in the first tertile in the crude model but not in the fully adjusted model including diabetes mellitus. In males, in the crude model, a significant association was found between TyG and CVM risk (HR = 1.60, 95% CI 1.00, 2.55; P for trend = 0.043) and adjustment for age strengthened it (HR = 1.91, 95% CI 1.20, 3.06; P for trend < 0.001) and other confounders adjustment did not change it substantially except for mediators in model 5 and diabetes mellitus in model 6 (HR = 1.01, 95% CI 0.56, 1.83; P for trend = 0.995). Non-cardiovascular mortality was related to the tertiles of TyG neither in females nor in males in any of the models.

**Table 3:** Multiple-adjusted HRs (and 95% CIs) for all-cause, CVD and non-CVD mortality across tertiles of triglyceride glucose index stratified by sex

All-cause mortality		Model 1	Model 2	Model 3	Model 4	Model 5	Model 6
Females	Tertile 1	1 (Ref.)	1 (Ref.)	1 (Ref.)	1 (Ref.)	1 (Ref.)	1 (Ref.)
	Tertile 2	1.49(1.04,2.14)	1.01(0.70, 1.45)	0.99(0.69, 1.42)	1.01(0.70, 1.45)	1.15(0.78, 1.68)	1.13(0.77, 1.66)
	Tertile 3	1.74(1.23,2.46)	1.14(0.80, 1.61)	1.11(0.79, 1.58)	1.14(0.80, 1.62)	1.37(0.94, 2.00)	1.18(0.79, 1.76)
	P trend	0.002	0.430	0.502	0.419	0.094	0.424
Males	Tertile 1	1 (Ref.)	1 (Ref.)	1 (Ref.)	1 (Ref.)	1 (Ref.)	1 (Ref.)
	Tertile 2	0.93(0.69, 1.25)	1.06(0.79, 1.44)	1.08(0.80, 1.46)	1.11(0.82, 1.50)	1.06(0.77, 1.45)	1.02(0.74, 1.41)
	Tertile 3	1.13(0.85,1.50)	1.34(1.01, 1.78)	1.39(1.04, 1.86)	1.42(1.06, 1.90)	1.38(0.99, 1.92)	1.06(0.74, 1.51)
	P trend	0.378	0.042	0.024	0.018	0.056	0.760
CVD mortality							
Females	Tertile 1	1 (Ref.)	1 (Ref.)	1 (Ref.)	1 (Ref.)	1 (Ref.)	1 (Ref.)
	Tertile 2	2.19(1.13, 4.25)	1.47(0.75,2.85)	1.45(0.75, 2.83)	1.44(0.74, 2.80)	1.39(0.69, 2.81)	1.35(0.67,2.74)
	Tertile 3	2.49(1.30,4.76)	1.63(0.85,3.13)	1.59(0.83, 3.03)	1.57(0.82, 3.02)	1.58(0.78, 3.18)	1.17(0.56,2.47)
	P trend	0.007	0.151	0.185	0.189	0.217	0.773
Males	Tertile 1	1 (Ref.)	1 (Ref.)	1 (Ref.)	1 (Ref.)	1 (Ref.)	1 (Ref.)
	Tertile 2	1.21(0.73, 1.99)	1.41(0.85,2.32)	1.45(0.88, 2.40)	1.51(0.91, 2.50)	1.30(0.76, 2.20)	1.20(0.70,2.04)
	Tertile 3	1.60(1.00, 2.55)	1.91(1.20,3.06)	1.90(1.18, 3.06)	1.96(1.21, 3.17)	1.58(0.92, 2.71)	1.01(0.56,1.83)
	P trend	0.043	< 0.001	0.008	0.006	0.099	0.995
Non-CVD mortality							
Females	Tertile 1	1 (Ref.)	1 (Ref.)	1 (Ref.)	1 (Ref.)	1 (Ref.)	1 (Ref.)
	Tertile 2	1.25(0.81, 1.93)	0.85(0.55, 1.31)	0.83(0.54, 1.29)	0.86(0.55, 1.32)	1.04(0.66, 1.66)	1.04(0.66, 1.65)
	Tertile 3	1.48(0.98, 2.24)	0.97(0.64, 1.47)	0.95(0.63, 1.44)	0.98(0.65, 1.49)	1.27(0.81, 2.01)	1.19(0.73, 1.92)
	P trend	0.063	0.955	0.894	0.988	0.271	0.471
Males	Tertile 1	1 (Ref.)	1 (Ref.)	1 (Ref.)	1 (Ref.)	1 (Ref.)	1 (Ref.)
	Tertile 2	0.80(0.55, 1.17)	0.91(0.62, 1.33)	0.91(0.62, 1.33)	0.93(0.64, 1.36)	0.94(0.63, 1.40)	0.93(0.62, 1.38)
	Tertile 3	0.91(0.64, 1.30)	1.08(0.75, 1.54)	1.15(0.80, 1.66)	1.16(0.80, 1.68)	1.28(0.84, 1.96)	1.11(0.71, 1.75)
	P trend	0.613	0.700	0.470	0.440	0.265	0.681

Model 1: crude

Model 2: adjusted for age (years)

Model 3: Additionally adjusted for education level (0–5 years/6–12 years/ > 12 years), marital status (married/not married), residency location (urban/rural)

Model 4: Additionally adjusted for global dietary index (GDI), smoking status (never have smoked/have ever smoked), and total daily physical activity (METs-min/day)

Model 5: Additionally adjusted for BMI (kg/m^2^), hypertension (yes/no), and high total cholesterol (yes/no)

Model 6: Additionally adjusted for diabetes mellitus (DM) (yes/no)

**Fig. 2:**
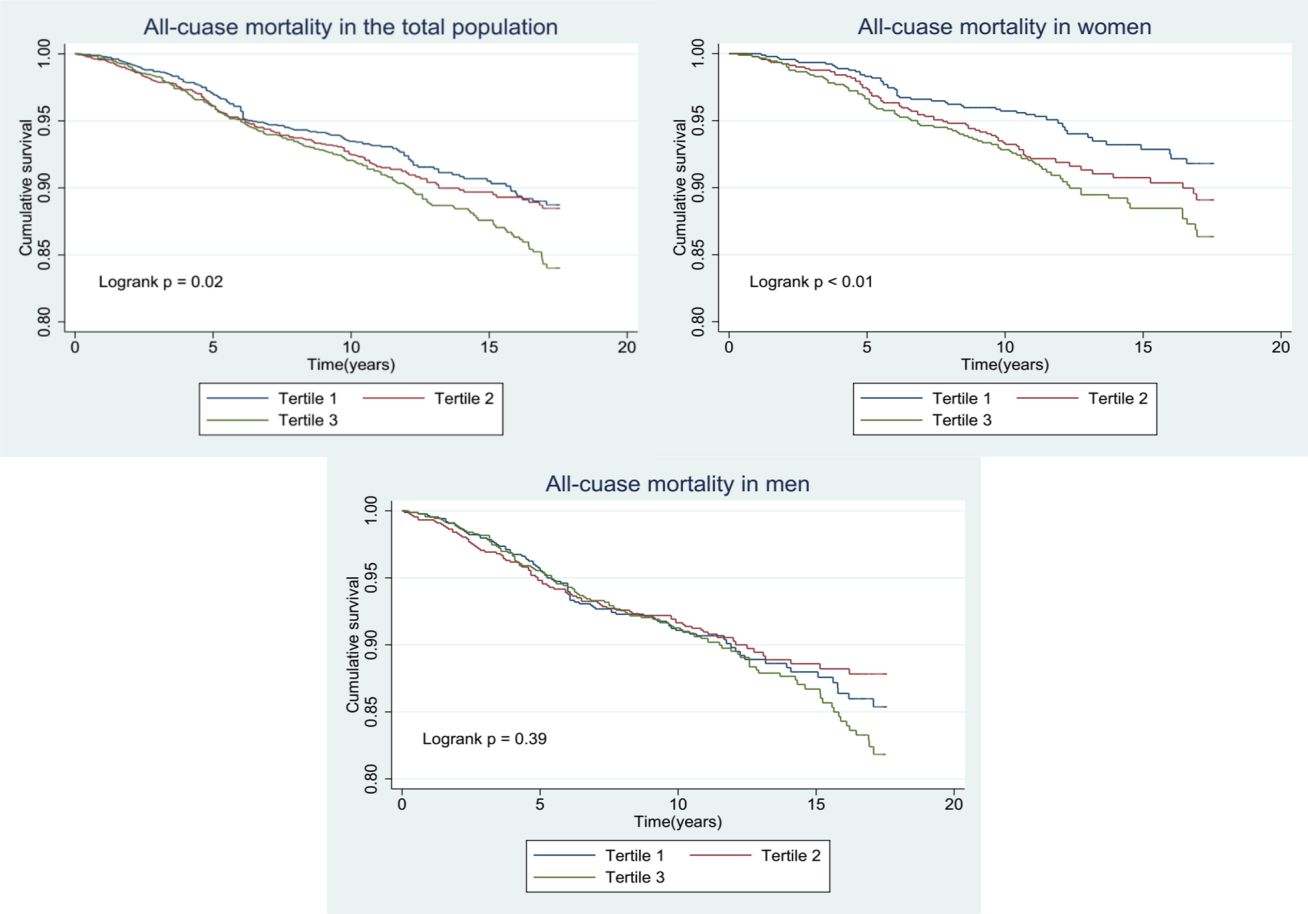
Kaplan–Meier curves to estimate cumulative hazard for the development of all-cause mortality by baseline triglyceride glucose index tertiles in total population, male participants, and female participants

**Fig. 3:**
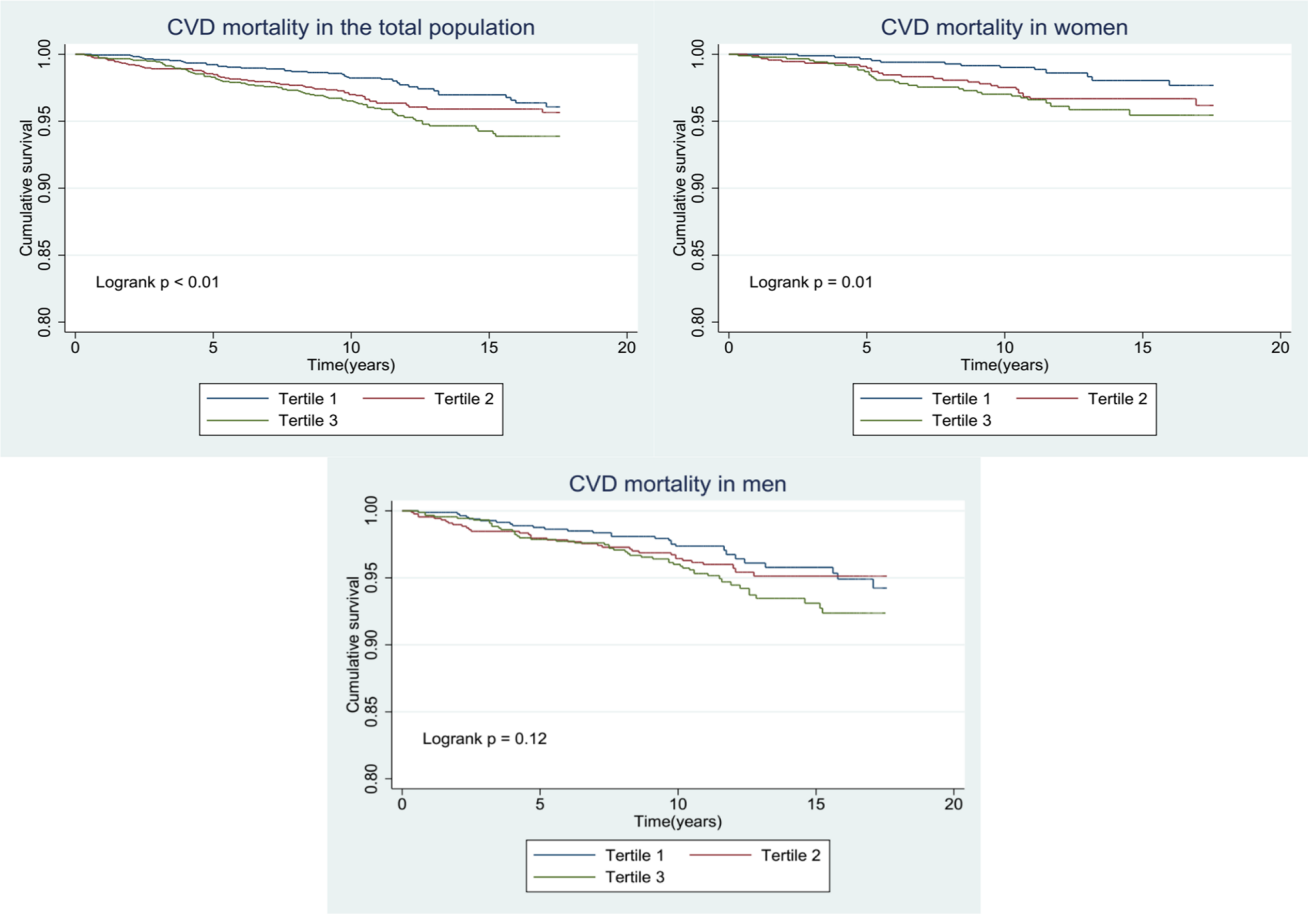
Kaplan–Meier curves to estimate cumulative hazard for the development of cardiovascular mortality by baseline triglyceride glucose index tertiles in **A** Total population, **B** Male, and **C** Female

**Fig. 4:**
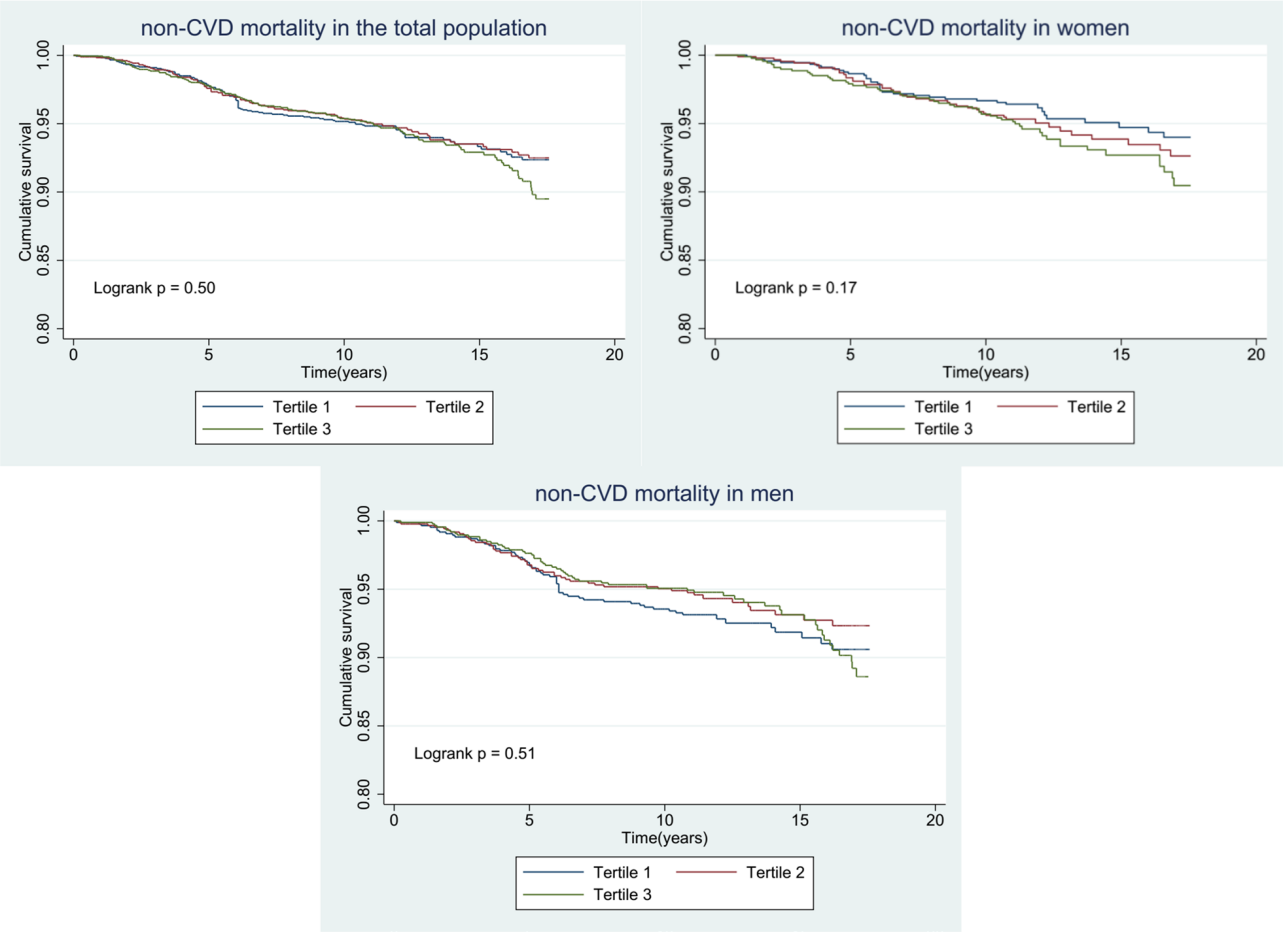
Kaplan–Meier curves to estimate cumulative hazard for the development of non-cardiovascular mortality by baseline triglyceride glucose index tertiles in **A** Total population, **B** Male, and **C** Female

Figures 2, 3, 4 display Kaplan–Meier survival analysis curves, illustrating the occurrence of ACM, CVM, and non-cardiovascular mortality, respectively across tertiles of TyG index in total population, men and women.

Additional file 1: Table S1 reveals the association of ACM, CVM and non-cardiovascular mortality with TG and FPG separately. Higher TG levels were not associated with increased risk of ACM and non-cardiovascular mortality in any of the model. However, although it was directly related to the risk of CVM, it was dependent on some mediators and disappeared after controlling their mediating effect. Regarding FPG, the higher FPG levels, the higher risk of ACM and CVM in all models except for the adjusted model for DM. Nevertheless, it was not related to the risk of non-cardiovascular mortality.

## Discussion

In the current cohort study, which involved 5432 Iranian adults from the general population, we observed a significant direct relationship between the baseline TyG index and CVM and ACM after 11.25 years of follow-up. This association was independent of various demographic, lifestyle and metabolic variables. Nevertheless, DM emerged as a significant confounding variable in the connection between TyG index and both CVM and ACM. In stratified analysis by sex, the increased risk of ACM and CVM with an increase in the TyG index was evident in males, but not females, though it depended on metabolic risk factors. Non-CVD mortality was not significantly related to the TyG index.

Atherosclerosis, type 2 DM, metabolic syndrome, and nonalcoholic fatty liver are just a few modern debilitating diseases that are thought to be triggered by IR [24]. Additionally, IR and ACM association in different populations and not only in diabetic patients has gained attention in recent years. TyG index, as an indicator of FPG and TG, is a more suitable indicator of IR state than the FPG and TG alone [25]. Prior research suggests that TyG index has a positive correlation with arterial stiffness [26], coronary artery calcification progression [27], DM incidents [28], LDL-C, TG, and estimated glomerular filtration rate [29]. All of which are traditional risk factors for CAD and this correlation can rationalize the prognostic value of the TyG index with CVM.

Our results are in line with previous studies regarding the increased rate of CVM and ACM among those with higher TyG index [29–32]. For example, after 7.6 years of follow-up, a higher TyG index was linked to an 18% greater risk of ACM in a study on 9254 American men over the age of 45 [32]. In another observational study on acute coronary syndrome patients with DM, individuals in the top tertile of the TyG index had an HR of 1.44 for ACM compared to the first tertile in a dose–response manner [29]. The precise predictive significance of TyG index regarding ACM is still controversial. In light of this, Lopez-Jaramillo et al. reported significant association between the higher TyG index and cardiovascular incidence and CVM, especially in LMIC in the PURE study. However, their results differ from ours as they did not find a significant association between the TyG index and non-cardiovascular mortality and ACM, while we observed an increased risk of ACM with higher TyG index [19]. Liu et al. also reported inconsistent results when it comes to ACM and CVM, and based on their systematic review and meta-analysis of 12 cohort studies, there was no association between the TyG index and ACM and CVM [6]. The inconsistent findings in various studies could be due to disparities in location and economic status. Our study of the Iranian population, which is considered a middle-income country, showed an increased risk for ACM and CVM. This may be explained by the fact that LMIC are more susceptible to insulin resistance, which is possibly caused by poor maternal nutrition resulting in low birthweight babies and exposure to obesogenic modern lifestyles in adulthood [19]. Additionally, as a result of habits developed through rapid urbanization leading to a sedentary lifestyle and consumption of high-energy foods, obesity and overweight rates are high among these populations [33]. Another key factor that can explain the heterogeneity between high income countries and LMIC is the level of education. The research suggests that people with lower levels of education in LMIC are at a significantly higher risk of experiencing serious cardiovascular events compared to those with higher levels of education [34].

Inconsistencies in findings regarding the impact of the TyG index on mortality with respect to sex have been documented previously [35–37]. Upon stratifying our outcomes by sex, we showed no statistically significant association between the TyG index and ACM or CVM in males and females after adjustment for metabolic mediators. However, in other adjusted models, the associations were evident in males, but not females. Sex differences found in our study was in line with another analysis in US adults which suggested the higher TyG, the higher mortality in males but not females [36]. Consistently, while higher TyG was a risk factor for atherosclerosis in only males in one study [38], some others failed to find a sex-specific association for various cardiovascular events [39, 40]. These variations may be attributed to an insufficient participant pool, underscoring the necessity for more expansive sex-specific studies with a larger participant cohort to precisely elucidate sex-based differences in the clinical implications of the TyG index. However, there is scarce data directly on TyG index and ACM and CVM. Exploring sex-specific association with respect to TyG and mortality is relevant due to the effect of sex on IR and mortality [41, 42].

More extensive observational and interventional studies are required to authenticate the effectiveness of using this index to forecast ACM and CVM in both the general population and diabetic patients. Nevertheless, the mechanism behind predictive value of the TyG index could be elaborated as follows. Firstly, increased mortality may be caused by several metabolic disorders including hyperglycemia, dyslipidemia, and HTN, all of which are strongly associated with a poor prognosis for CVD events [43]. Secondly, IR may be associated with enhanced oxidative stress, inflammatory response, impaired endothelial function, and consequently greater vascular lesion [44]. Moreover, IR and the state of chronic hyperinsulinemia lead to increased sodium retention and sympathetic activity, which in turn raise blood pressure and renal and vascular damage [45]. And lastly, chronic illnesses have been observed to be more prevalent among patients with higher TyG index which can further complicate patients’ conditions [32]. Although FPG was a strong predictor of CVM in our analysis, for ACM, its predictive value was considerably affected by adjustment for confounders and therefore TyG might outweigh FPG to predict ACM.

The main strength of our study was that we confirmed that predicting value of high TyG index for mortality from all-cause and CVD in the general Iranian population was dependent on the status of diabetes. Other strengths of our investigation included the large number of people who participated, the prospective design, and the long follow-up period. Another supremacy of our study was that we included participants from both rural and urban areas of Iran and therefore our results can be extrapolated to similar areas in the country. Moreover, as highlighted by previous PURE studies [19, 37], fundamental differences exist between LMIC and high-income countries regarding risk factors for cardiovascular disease outcomes and mortality. This contextual specificity adds a novel dimension to our investigation. Finally, our analysis delves into the sex-specific differences of the TyG index, contributing to the ongoing discourse in the literature. Our study, however, had a number of flaws. The TyG index could not be compared to other IR gold standard measurements, such as the HOMA-IR and HIEC, duet to the lack of measurements for fasting plasma insulin. Additionally, residual confounding factors might have an impact on the patients’ prognosis. Finally, we only measured the baseline TyG index and did not evaluate this index throughout the years and its transition.

In summary, the predicting value of TyG index for ACM and CVM might be mediated by diabetes status. Further comprehensive investigations are imperative to ascertain the precise association of the TyG index with mortality and to delineate whether diabetes plays a mediator role in this relationship. This may be considered a promising aspect of using TyG index in clinical practice. Due to its feasibility, this index has the potential to aid in detecting individuals at increased risk for developing cardiovascular events and mortality early on.

### Supplementary Information

**Additional file 1: Table S1.** Multiple-adjusted HRs (and 95% CIs) for triglyceride and fasting blood sugar and all-cause, CVD and non-CVD mortality.

## Data Availability

All data generated or analyzed during this study are included in the published article titled, the Isfahan cohort study rationale, methods, and main findings [20].

## Abbreviations

CVD: Cardiovascular disease
CVM: Cardiovascular mortality
ACM: All-cause mortality
TyG index: Triglyceride glucose index
CAD: Coronary artery disease
PAD: Peripheral artery disease
HIEC: Hyper insulinemic-euglycemic
HOMA-IR: Hemostasis model assessment of insulin resistance
SBP: Systolic blood pressure
DBP: Diastolic blood pressure
DM: Diabetes mellitus
HTN: Hypertension
FPG: Fasting plasma glucose
TG: Triglyceride
HDL-c: High density lipoprotein cholesterol
GDI: Global dietary index

## References

[1] Riaz H, Khan MS, Siddiqi TJ, Usman MS, Shah N, Goyal A (2018). Association between obesity and cardiovascular outcomes: a systematic review and meta-analysis of Mendelian randomization studies. JAMA Netw Open.

[2] Salari N, Doulatyari PK, Daneshkhah A, Vaisi-Raygani A, Jalali R, Jamshidi PK (2020). The prevalence of metabolic syndrome in cardiovascular patients in Iran: a systematic review and meta-analysis. Diabetol Metab Syndr.

[3] Liao Y, Zhang R, Shi S, Zhao Y, He Y, Liao L (2022). Triglyceride-glucose index linked to all-cause mortality in critically ill patients: a cohort of 3026 patients. Cardiovasc Diabetol.

[4] Mohammad NS, Nazli R, Zafar H, Fatima S (2022). Effects of lipid based multiple micronutrients supplement on the birth outcome of underweight pre-eclamptic women: a randomized clinical trial. Pak J Med Sci.

[5] Zhang X, Li J, Zheng S, Luo Q, Zhou C, Wang C (2017). Biosci Rep.

[6] Liu X, Tan Z, Huang Y, Zhao H, Liu M, Yu P (2022). Relationship between the triglyceride-glucose index and risk of cardiovascular diseases and mortality in the general population: a systematic review and meta-analysis. Cardiovasc Diabetol.

[7] Che B, Zhong C, Zhang R, Pu L, Zhao T, Zhang Y (2023). Triglyceride-glucose index and triglyceride to high-density lipoprotein cholesterol ratio as potential cardiovascular disease risk factors: an analysis of UK biobank data. Cardiovasc Diabetol.

[8] Xiong S, Chen Q, Long Y, Su H, Luo Y, Liu H (2023). Association of the triglyceride-glucose index with coronary artery disease complexity in patients with acute coronary syndrome. Cardiovasc Diabetol.

[9] Caliskan S, Boyuk F (2023). Is triglyceride-glucose index a valuable parameter in peripheral artery disease?. Cureus.

[10] Liao C, Xu H, Jin T, Xu K, Xu Z, Zhu L (2022). Triglyceride-glucose index and the incidence of stroke: a meta-analysis of cohort studies. Front Neurol.

[11] Zhou D, Liu X-C, Kenneth L, Huang Y-Q, Feng Y-Q (2022). A non-linear association of triglyceride glycemic index with cardiovascular and all-cause mortality among patients with hypertension. Front Cardiovasc Med.

[12] Yusuf S, Rangarajan S, Teo K, Islam S, Li W, Liu L (2014). Cardiovascular risk and events in 17 low-, middle-, and high-income countries. N Engl J Med.

[13] Tabrizi JS, Sadeghi-Bazargani H, Farahbakhsh M, Nikniaz L, Nikniaz Z (2018). Prevalence and associated factors of overweight or obesity and abdominal obesity in Iranian population: a population-based study of Northwestern Iran. Iran J Public Health.

[14] Mazloomzadeh S, Rashidi Khazaghi Z, Mousavinasab N (2018). The prevalence of metabolic syndrome in Iran: a systematic review and meta-analysis. Iran J Public Health.

[15] Khodakarami R, Abdi Z, Ahmadnezhad E, Sheidaei A, Asadi-Lari M (2022). Prevalence, awareness, treatment and control of diabetes among Iranian population: results of four national cross-sectional STEPwise approach to surveillance surveys. BMC Public Health.

[16] Coll JL, del Bibiloni MM, Salas R, Pons A, Tur JA (2015). Prevalence and related risk factors of overweight and obesity among the adult population in the Balearic Islands, a Mediterranean Region. Obesity facts.

[17] Jafari-Adli S, Jouyandeh Z, Qorbani M, Soroush A, Larijani B, Hasani-Ranjbar S (2014). Prevalence of obesity and overweight in adults and children in Iran; a systematic review. J Diabetes Metab Disord.

[18] Sarrafzadegan N, Mohammmadifard N (2019). Cardiovascular disease in iran in the last 40 years: prevalence, mortality, morbidity, challenges and strategies for cardiovascular prevention. Arch Iran Med.

[19] Lopez-Jaramillo P, Gomez-Arbelaez D, Martinez-Bello D, Abat MEM, Alhabib KF, Avezum Á (2023). Association of the triglyceride glucose index as a measure of insulin resistance with mortality and cardiovascular disease in populations from five continents (PURE study): a prospective cohort study. Lancet Healthy Longev.

[20] Sarrafzadegan N, Talaei M, Sadeghi M, Kelishadi R, Oveisgharan S, Mohammadifard N (2011). The Isfahan cohort study: rationale, methods and main findings. J Hum Hypertens.

[21] Iida S, Osawa S, Yonemitsu H (1994). A new precipitation method with magnetic separation for high-density-lipoprotein cholesterol assay. Clin Chim Acta.

[22] American Diabetes Association. Standards of Medical Care in Diabetes-2022 Abridged for Primary Care Providers. Clin Diabetes. 2022; 40(1): 10–38. 10.2337/cd22-as01 PMC8865785 35221470

[23] Guerrero-Romero F, Simental-Mendía LE, González-Ortiz M, Martínez-Abundis E, Ramos-Zavala MG, Hernández-González SO (2010). The product of triglycerides and glucose, a simple measure of insulin sensitivity. Comparison with the euglycemic-hyperinsulinemic clamp. J Clin Endocrinol Metab.

[24] Lee SH, Park SY, Choi CS (2022). Insulin resistance: from mechanisms to therapeutic strategies. Diabetes Metab J.

[25] Simental-Mendía LE, Rodríguez-Morán M, Guerrero-Romero F (2008). The product of fasting glucose and triglycerides as surrogate for identifying insulin resistance in apparently healthy subjects. Metab Syndr Relat Disord.

[26] Wu S, Xu L, Wu M, Chen S, Wang Y, Tian Y (2021). Association between triglyceride-glucose index and risk of arterial stiffness: a cohort study. Cardiovasc Diabetol.

[27] Park K, Ahn CW, Lee SB, Kang S, Nam JS, Lee BK (2019). Elevated TyG index predicts progression of coronary artery calcification. Diabetes Care.

[28] Pranata R, Huang I, Lim MA, Vania R (2021). The association between triglyceride-glucose index and the incidence of type 2 diabetes mellitus—a systematic review and dose–response meta-analysis of cohort studies. Endocrine.

[29] Shen J, Feng B, Fan L, Jiao Y, Li Y, Liu H (2023). Triglyceride glucose index predicts all-cause mortality in oldest-old patients with acute coronary syndrome and diabetes mellitus. BMC Geriatr.

[30] Liu X-C, He G-D, Lo K, Huang Y-Q, Feng Y-Q (2021). The triglyceride-glucose index, an insulin resistance marker, was non-linear associated with all-cause and cardiovascular mortality in the general population. Front Cardiovasc Med.

[31] Zhang Y, Ding X, Hua B, Liu Q, Gao H, Chen H (2021). Predictive effect of triglyceride-glucose index on clinical events in patients with type 2 diabetes mellitus and acute myocardial infarction: results from an observational cohort study in China. Cardiovasc Diabetol.

[32] Sun M, Guo H, Wang Y, Ma D (2022). Association of triglyceride glucose index with all-cause and cause-specific mortality among middle age and elderly US population. BMC Geriatr.

[33] Lopez-Jaramillo P, Lahera V, Lopez-Lopez J (2011). Epidemic of cardiometabolic diseases: a Latin American point of view. Ther Adv Cardiovasc Dis.

[34] Rosengren A, Smyth A, Rangarajan S, Ramasundarahettige C, Bangdiwala SI, AlHabib KF (2019). Socioeconomic status and risk of cardiovascular disease in 20 low-income, middle-income, and high-income countries: the Prospective Urban Rural Epidemiologic (PURE) study. Lancet Glob Health.

[35] Chen J, Wu K, Lin Y, Huang M, Xie S (2023). Association of triglyceride glucose index with all-cause and cardiovascular mortality in the general population. Cardiovasc Diabetol.

[36] Yu Y, Wang J, Ding L, Huang H, Cheng S, Deng Y (2023). Sex differences in the nonlinear association of triglyceride glucose index with all-cause and cardiovascular mortality in the general population. Diabetol Metab Syndr.

[37] Zhou D, Liu X-C, Kenneth L, Huang Y-Q, Feng Y-Q (2022). A non-linear association of triglyceride glycemic index with cardiovascular and all-cause mortality among patients with hypertension. Front Cardiovasc Med.

[38] Lu YW, Chang CC, Chou RH, Tsai YL, Liu LK, Chen LK (2021). Gender difference in the association between TyG index and subclinical atherosclerosis: results from the I-Lan Longitudinal Aging Study. Cardiovasc Diabetol.

[39] Yang K, Liu W (2021). Triglyceride and glucose index and sex differences in relation to major adverse cardiovascular events in hypertensive patients without diabetes. Front Endocrinol (Lausanne).

[40] Hong S, Han K, Park CY (2020). The triglyceride glucose index is a simple and low-cost marker associated with atherosclerotic cardiovascular disease: a population-based study. BMC Med.

[41] Kautzky-Willer A, Harreiter J, Pacini G (2016). Sex and gender differences in risk, pathophysiology and complications of type 2 diabetes mellitus. Endocr Rev.

[42] GBD 2017 Causes of Death Collaborators. Global, regional, and national age-sex-specific mortality for 282 causes of death in 195 countries and territories, 1980–2017: a systematic analysis for the Global Burden of Disease Study 2017. Lancet. 2018; 392(10159):1736–88. 10.1016/S0140-6736(18)32203-7 PMC6227606 30496103

[43] Zhang R, Shi S, Chen W, Wang Y, Lin X, Zhao Y (2023). Independent effects of the triglyceride-glucose index on all-cause mortality in critically ill patients with coronary heart disease: analysis of the MIMIC-III database. Cardiovasc Diabetol.

[44] Gao S, Ma W, Huang S, Lin X, Yu M (2021). Impact of triglyceride-glucose index on long-term cardiovascular outcomes in patients with myocardial infarction with nonobstructive coronary arteries. Nutr Metab Cardiovasc Dis.

[45] da Silva AA, do Carmo JM, Li X, Wang Z, Mouton AJ, Hall JE (2020). Role of hyperinsulinemia and insulin resistance in hypertension: metabolic syndrome revisited. Can J Cardiol.

