# Supplementary material for: Association of the triglyceride glucose index with all-cause and cardiovascular mortality in a general population of Iranian adults
Source: Cardiovasc Diabetol. 2024 Feb 12;23:66. doi: 10.1186/s12933-024-02148-8 (PMC10863153; doi:10.1186/s12933-024-02148-8)
Supplement: Supplementary file 1 — Additional file 1: Table S1. Multiple-adjusted HRs (and 95% CIs) for triglyceride and fasting blood sugar and all-cause, CVD and non-CVD mortality. [file 12933_2024_2148_MOESM1_ESM.docx]

Additional file Table 1. Multiple-adjusted HRs (and 95% CIs) for triglyceride and fasting blood sugar and all-cause, CVD and non-CVD mortality.

| **All-cause mortality** | | **Model 1** | **Model 2** | **Model 3** | **Model 4** | **Model 5** | **Model 6** |
| --- | --- | --- | --- | --- | --- | --- | --- |
|  |  |  |  |  |  |  |  |
| **TG** | Tertile 1 | 1 (Ref.) | 1 (Ref.) | 1 (Ref.) | 1 (Ref.) | 1 (Ref.) | 1 (Ref.) |
|  | Tertile 2 | 1.10 (0.88, 1.37) | 1.03 (0.83, 1.29) | 1.03 (0.83, 1.29) | 1.04 (0.83, 1.29) | 1.05 (0.83, 1.32) | 1.03 (0.82, 1.30) |
|  | Tertile 3 | 1.09 (0.87, 1.35) | 1.06 (0.85, 1.32) | 1.07 (0.86, 1.33) | 1.08 (0.86, 1.35) | 1.10 (0.86, 1.42) | 1.01 (0.78, 1.30) |
|  | P trend | 0.468 | 0.605 | 0.557 | 0.510 | 0.438 | 0.947 |
|  |  |  |  |  |  |  |  |
| FPG | Tertile 1 | 1 (Ref.) | 1 (Ref.) | 1 (Ref.) | 1 (Ref.) | 1 (Ref.) | 1 (Ref.) |
|  | Tertile 2 | 1.11 (0.87, 1.41) | 1.12 (0.88, 1.43) | 1.13 (0.89, 1.44) | 1.17 (0.92, 1.49) | 1.19 (0.93, 1.52) | 1.19 (0.94, 1.52) |
|  | Tertile 3 | 1.73 (1.39, 2.16) | 1.33 (1.06, 1.65) | 1.32 (1.06, 1.64) | 1.35 (1.09, 1.69) | 1.38 (1.10, 1.74) | 1.12 (0.87, 1.43) |
|  | P trend | <0.001 | 0.010 | 0.013 | 0.007 | 0.005 | 0.378 |
| **CVD mortality** |  |  |  |  |  |  |  |
| **TG** | Tertile 1 | 1 (Ref.) | 1 (Ref.) | 1 (Ref.) | 1 (Ref.) | 1 (Ref.) | 1 (Ref.) |
|  | Tertile 2 | 1.74 (1.18, 2.55) | 1. 67 (1.14, 2.46) | 1.66 (1.13, 2.45) | 1.68 (1.14, 2.47) | 1.56 (1.04, 2.33) | 1.53 (1.03, 2.29) |
|  | Tertile 3 | 1.64 (1.11, 2.42) | 1.65 (1.12, 2.44) | 1.64 (1.11, 2.42) | 1.65 (1.11, 2.43) | 1.42 (0.92, 2.19) | 1.23 (0.79, 1.90) |
|  | P trend | 0.017 | 0.015 | 0.017 | 0.016 | 0.158 | 0.462 |
|  |  |  |  |  |  |  |  |
| FPG | Tertile 1 | 1 (Ref.) | 1 (Ref.) | 1 (Ref.) | 1 (Ref.) | 1 (Ref.) | 1 (Ref.) |
|  | Tertile 2 | 1.30 (0.85, 1.97) | 1.32 (0.87, 2.00) | 1.32 (0.87, 2.00) | 1.41 (0.92, 2.14) | 1.41 (0.92, 2.17) | 1.42 (0.93, 2.18) |
|  | Tertile 3 | 2.33 (1.60, 3.39) | 1.79 (1.23, 2.61) | 1.76 (1.21, 2.57) | 1.84 (1.26, 2.69) | 1.75 (1.18, 2.59) | 1.21 (0.79, 1.87) |
|  | P trend | <0.001 | 0.002 | 0.003 | 0.001 | 0.005 | 0.412 |
| **Non-CVD mortality** |  |  |  |  |  |  |  |
| **TG** | Tertile 1 | 1 (Ref.) | 1 (Ref.) | 1 (Ref.) | 1 (Ref.) | 1 (Ref.) | 1 (Ref.) |
|  | Tertile 2 | 0.86 (0.66, 1.13) | 0.80 (0.61, 1.06) | 0.81 (0.61, 1.06) | 0.81 (0.61, 1.06) | 0.86 (0.64, 1.14) | 0.84 (0.63, 1.13) |
|  | Tertile 3 | 0.88 (0.67, 1.15) | 0.84 (0.64, 1.11) | 0.86 (0.65, 1.13) | 0.86 (0.66, 1.14) | 0.98 (0.72, 1.33) | 0.93 (0.68, 1.27) |
|  | P trend | 0.351 | 0.224 | 0.266 | 0.290 | 0.882 | 0.625 |
|  |  |  |  |  |  |  |  |
| FPG | Tertile 1 | 1 (Ref.) | 1 (Ref.) | 1 (Ref.) | 1 (Ref.) | 1 (Ref.) | 1 (Ref.) |
|  | Tertile 2 | 1.03 (0.77, 1.38) | 1.04 (0.77, 1.39) | 1.05 (0.78, 1.41) | 1.08 (0.80, 1.45) | 1.10 (0.82, 1.49) | 1.11 (0.82, 1.49) |
|  | Tertile 3 | 1.47 (1.12, 1.92) | 1.12 (0.85, 0.47) | 1.12 (0.85, 1.47) | 1.14 (0.87, 1.50) | 1.23 (0.93, 1.63) | 1.09 (0.80, 1.47) |
|  | P trend | 0.004 | 0.408 | 0.417 | 0.342 | 0.149 | 0.577 |

Model 1: crude.

Model 2: adjusted for age (years) and sex (man/woman)

Model 3: Additionally adjusted for education level (0-5 years/6-12 years/>12 years), marital status (married/not married), residency location (urban/rural)

Model 4: Additionally adjusted for global dietary index (GDI), smoking status (never have smoked/have ever smoked), and total daily physical activity (METs-min/day)

Model 5: Additionally adjusted for BMI (kg/m^2^), hypertension (yes/no), and high total cholesterol (yes/no)

Model 6: Additionally adjusted for diabetes mellitus (DM) (yes/no)
